# Prognostic factors of total hip replacement during a 2-year period in participants enrolled in supervised education and exercise therapy: a prognostic study of 3657 participants with hip osteoarthritis

**DOI:** 10.1186/s13075-021-02608-6

**Published:** 2021-09-07

**Authors:** Stine Clausen, Jan Hartvigsen, Eleanor Boyle, Ewa M. Roos, Dorte Thalund Grønne, Martin Thomsen Ernst, Bodil Arnbak, Søren T. Skou

**Affiliations:** 1 Center for Muscle and Joint Health, Department of Sports Science and Clinical Biomechanics, University of Southern Denmark, Campusvej 55, 5230 Odense M, Denmark; 2 Department of Radiology, Hospital Lillebælt, Vejle, Denmark; 3 Chiropractic Knowledge Hub, Odense, Denmark; 4 Clinical Pharmacology, Pharmacy and Environmental Medicine, Department of Public Health, University of Southern Denmark, Odense, Denmark; 5 The Research Unit PROgrez, Department of Physiotherapy and Occupational Therapy, Næstved-Slagelse-Ringsted Hospitals, Slagelse, Region Zealand Denmark

**Keywords:** Hip osteoarthritis, Prediction, Prognostic model, Total hip replacement

## Abstract

**Background:**

Evidence on prognostic factors associated with progression to total hip replacement (THR) in hip osteoarthritis (OA) is for the most patient- and disease-specific characteristics either conflicting or inconclusive. Therefore, the objectives of this study of participants with hip OA enrolled in a structured program of supervised education and exercise therapy were to describe the rate of THR and to identify prognostic factors for receiving THR within the following 2 years.

**Methods:**

Participants aged ≥ 45 years with hip OA enrolled in Good Life with osteoArthritis in Denmark (GLA:D®) from July 2014 to March 2017 were included. Potential prognostic factors included demographic and disease-specific baseline characteristics and measures of physical activity and quality of life (QoL). Information on THR was retrieved from The Danish National Patient Registry. A multivariable Cox proportional hazards model was developed.

**Results:**

Of 3657 included participants, 30% received a THR within 2 years. Of the 100 participants already wait-listed for THR, 60% had the procedure. Of 22 candidate prognostic factors, 14 were statistically significant for receiving THR. Factors associated with a faster rate of THR included being “male” (HR 1.43), having “self-reported radiographic hip OA” (HR 2.32), being “wait-listed for THR” (HR 2.17), and having a higher “pain intensity” (HR 1.01). In contrast, faster “walking speed” (HR 0.64), better “hip-related QoL” (HR 0.98), and having “three or more comorbidities” (HR 0.62) were predictive of a slower rate of THR.

**Conclusion:**

During the 2-year follow-up period, 30% of the cohort received a THR. Notably, 40% of those wait-listed for THR when entering the program did not receive THR within 2 years. A number of baseline prognostic factors for receiving THR were identified.

**Supplementary Information:**

The online version contains supplementary material available at 10.1186/s13075-021-02608-6.

## Background

Osteoarthritis (OA) affects more than 500 million people worldwide and is a major cause of disability [1], with hip OA being one of the most common and disabling [2]. Clinical guidelines universally recommend patient education and exercise therapy as first-line treatments for hip OA [3, 4]. Total hip replacement surgery (THR) should only be considered for patients who experience joint symptoms (pain, stiffness, and reduced function) with a substantial impact on their quality of life (QoL) and who are not responding well to non-surgical treatments [5].

A recent systematic review [6] summarized the evidence on patient- and disease-specific factors associated with progression in patients with hip OA and found strong evidence that more pronounced radiographic changes were associated with faster progression to THR. However, for most demographic and clinical characteristics, the findings were either conflicting or inconclusive. The authors concluded that more high-quality research into prognostic factors for hip OA is needed. Knowledge gained from such research can benefit patients because it helps clinicians better inform patients about their prognosis and guide treatment decisions.

Therefore, the objectives of this study of participants with hip OA enrolled in a structured program of supervised education and exercise therapy were: (1) to describe the rate of THR and (2) to identify prognostic factors, collected at the time of enrolment, for receiving THR within the following 2 years.

## Method

The TRIPOD guideline for Transparent Reporting of a multivariable prediction model for Individual Prognosis Or Diagnosis [7] was followed to report this prospective cohort study using data from the ongoing nationwide initiative “Good Life with osteoArthritis in Denmark” (GLA:D®) [8] and The Danish National Patient Registry [9].

### Data source and patients

Participants enrolled in GLA:D® from July 1, 2014, to March 1, 2017, were included in the study. GLA:D® is an evidence-based non-surgical treatment program provided to people with symptoms associated with knee or hip OA and consists of two education sessions and 12 sessions of supervised neuromuscular exercises delivered in primary care settings by trained physiotherapists. A detailed description of GLA:D® is provided elsewhere [8, 10]. Patients are typically referred to the GLA:D® program by their general practitioner or an orthopedic surgeon. If referred from the general practitioner, approx. 40% of the treatment cost is reimbursed, and by referral from an orthopedic surgeon, the total treatment cost is reimbursed. Patients can also refer themselves directly to the physiotherapist but would then have to pay the entire treatment cost. Upon enrolling in the program, participants and physiotherapists fill in electronic questionnaires, and data are stored in an electronic registry. In this study, baseline data were linked to national registries on an individual level via the unique central person registry number assigned to all persons residing in Denmark. The Danish National Patient Registry contains information on diagnoses and procedures performed at all hospitals in Denmark. If THR is performed, a surgical code and a date for the procedure are linked to the patient’s central person registry number. Death or emigration before any surgery was considered censoring events, and these data were extracted from the Danish Civil Registration System. The registries are considered to have high data quality and completeness [9, 11].

The time period for data collection was determined by the presence of variables in the database (several of the candidate prognostic factors that we found relevant were introduced in the database in 2014) and the possibility to pair with registry data from The Danish National Patient Registry that was available up until March 1, 2019. The follow-up duration of 2 years was chosen as we wanted to investigate prognostic factors for progression to THR in the first years after enrolling in the program.

Participants who had completed the baseline questionnaire were included if they were 45 years or older, had a primary complaint of hip pain, and were excluded if they reported THR in the index hip at baseline or had incomplete data for any candidate prognostic factors.

### Outcome

The outcome was having a THR within the 2-year follow-up period and was retrieved via surgical codes from The Danish National Patient Registry.

### Prognostic factors

Potential prognostic factors were selected based on previously published studies [6, 12, 13] and compared to data available in the GLA:D® registry and the authors’ expert opinions. The potential prognostic factors included age, sex, body mass index (BMI), smoking, living alone, sick leave, educational level, employment status, self-reported radiographic OA, whether wait-listed for THR, previous joint replacement in the other hip or knees, comorbidities, use of pain medication, fear of joint damage from physical activity, bilateral hip symptoms, number of painful areas during the last 24 h, hip pain (VAS), University of California, Los Angeles Physical Activity Scale, Hip disability and Osteoarthritis Outcome Score (quality of life subscale score), Arthritis Self-Efficacy Scale, The EuroQoL 5-Dimensions 5-Level questionnaire 40 m walk test (m/s), and 30 s chair stand test (number of rises). The factors are further described in Table 1, which also includes the coding of the factors. The data on whether the participants were on a waiting list for THR was registered by the therapist. To answer yes, the patients should have consulted a surgeon. The variable “duration of symptoms” had a high number of missing values (24%) due to a technical problem during data collection. Therefore, and because the recent systematic review [6] found moderate evidence for no association with THR, it was not included as a potential prognostic factor. The two tests of physical function, the 40 m fast-paced walking test and the 30 s chair-stand tests [14] were conducted under the supervision of a physiotherapist.

**Table 1:** Candidate prognostic factors for participants included in the study (*n* = 3657)

Variables		Coding for analysis
Age, mean (SD)	66.5 (8.6)	Continuous
Sex, female, *n* (%)	2687 (73%)	Categorical
BMI, kg/m^2^, mean (SD)	26.9 (4.7)	Continuous
Current smoking, *n* (%)	353 (9.7%)	Categorical
Living alone, *n* (%)	1006 (28%)	Categorical
Sick leave due to hip problems for more than one month during the past 12 months, *n* (%)	93 (2.5%)	Categorical
Educational level, *n* (%) Primary and lower secondary school (9–10 years) Higher general examination program (12-13 years) Short-cycle higher education (less than three years more) Medium-cycle higher education (three to four years more) Long-cycle higher education (minimum five years more)	620 (17%) 372 (10%) 665 (18%) 1550 (42%) 450 (12%)	Categorical
Employment, *n* (%) Employed/student Unemployed Retired Self-imposed early retirement Early retirement due to low ability to work On sick leave full time or part time	1026 (28%) 49 (1.3%) 2131 (58%) 222 (6.1%) 119 (3.3%) 110 (3.0%)	Categorical Collapsed as either “no” regardless of what kind of retirement or “yes” regardless of current sick leave or temporary unemployment.
Self-reported radiographic OA, *n* (%) Had x-ray with radiographic OA Had x-ray without radiographic OA Had no x-ray or do not know	3007 (82%) 131 (3.6%) 519 (14%)	Categorical Collapsed as either “present” (had x-ray with radiographic OA) or “absent” (had x-ray with no radiographic OA, had no x-ray or do not know).
Wait-listed for THR of the index hip, *n* (%)	100 (2.7%)	Categorical
Joint replacement in the other hip or knees, *n* (%)	362 (9.9%)	Categorical
Comorbidities, *n* (%) None One Two Three or more	1425 (39%) 1321 (36%) 616 (17%) 295 (8%)	Categorical
Pain medication the last 3 months, *n* (%) No use of pain medication Has used only paracetamol/acetaminophen and/or NSAID Has used opioids (everyone using opioids also used paracetamol/acetaminophen and/or NSAID)	1308 (36%) 2033 (56%) 316 (8.6%)	Categorical: Collapsed into pain medication usage in the past 3 months “yes” (used paracetamol, acetaminophen, NSAID or opioids) or “no” (did not use any type of pain medication)
Fear of joint damage from physical activity, *n* (%)	365 (10%)	Categorical
Bilateral hip symptoms, *n* (%)	946 (26%)	Categorical
Number of painful areas during the last 24 h (0–56 areas market on a body chart front and rear view), median (IQR)	3 (3)	Continuous
Hip pain (average) during the last month (VAS 0–100), median (IQR)	48 (21)	Continuous
Duration of symptoms in the index joint, months (*n* = 2770), median (IQR)	24 (40)	Not included as a prognostic factor
UCLA activity score (1–10), median (IQR)	6 (3)	Categorical: In the analysis, the original 10 categories were collapsed into 5
HOOS quality of life subscale score, median (IQR) (0–100)	50 (19)	Continuous
Self-Efficacy (ASES) median (IQR) (10–100)	68 (26)	Continuous
Health-related quality of life (EQ-5D-5L), median (IQR) (− 0.624 to 1)	0.723 (0.110)	Continuous
40 m walk test (m/s), median (IQR)	1.49 (0.43)	Continuous
30 s chair stand test (no. of rises), median (IQR)	12 (5)	Continuous

UCLA, University of California, Los Angeles Physical Activity Scale (1–10). Level 10 is very high and 1 is very low. HOOS QoL, Hip disability and Osteoarthritis Outcome Score, the quality-of-life subscale score (0–100 worst to best).

ASES, Arthritis Self-Efficacy Scale. Only subscales for pain and other symptoms were collected and a mean were calculated. Higher scores indicate higher self-efficacy. EQ-5D-5L, The EuroQoL 5-Dimensions 5-Level questionnaire presented as an index value scored using the Danish crosswalk value set

The wording of the questions in the GLA:D® registry, response options, scoring, and coding methods can be found in Additional file 1.

### Statistical analysis

Statistical analysis was performed using Stata 16.1 (StataCorp, College Station, TX, USA).

The proportion of participants receiving THR within the study period was calculated. Kaplan-Meier curves were used to describe the rate of THR. After 2 years, if participants had not received THR, they were censored. Censoring could also be due to death or emigration during the study period.

#### Model building

A multivariable Cox proportional hazards model for receiving THR during the study period was developed. We used age as the timescale, which allows for a nonparametric age effect [15], and is appropriate for long-term studies in which subjects’ age, rather than the time they have been in the study, is likely to be the most crucial determinant of their risk of disease.

Model development and validation were performed in five steps [16]:

1. Prior to analyses, candidate prognostic factors were investigated for multicollinearity and correlations. Multicollinearity was investigated by calculating variance inflation factors (VIFs). The level of multicollinearity was not considered problematic if the mean VIF was ≤ 2 and individual VIFs were ≤ 4 [17]. A pairwise correlation (Spearman) of *r* ≥ 0.7 was deemed too high for regression analysis, and the most clinically relevant and easily obtained variable was chosen for the model.
2. A univariate Cox regression model was fitted for each candidate prognostic factor, and proportionality was checked using Schoenfeld residuals. The cumulative sums of martingale residuals were used to assess the continuous candidate prognostic factors’ linearity. The unadjusted estimates were not used to screen variables for entry into the multivariable model, as this is not recommended [18].
3. A multivariable Cox regression model was developed using backward stepwise elimination [16]. Candidate prognostic factors with *p* values < .05 were excluded one by one, excluding those with the highest *p* values first. A likelihood-ratio test of the model fit was performed at each step, comparing the reduced model to the previous model. If the model fit was not significantly different for the two models, the candidate prognostic factor was excluded.
4. The final model’s beta estimates were bootstrapped (1000 samples) to check the internal validity of the model. When the 95% confidence intervals for the hazard rates in the final and the bootstrapped models are overlapping the risk of overfitting is low.
5. The model’s performance in terms of discrimination was assessed using Harrell’s c-index [19]. A model with a c-index of 0.5 has no predictive ability while a c-index of 1.0 indicates perfect predictive ability.

#### Sample size and missing data

A large cohort (3657) and the number of participants receiving THR (1114) enabled the investigation of the 22 candidate prognostic factors without the risk of overfitting [7].

Only participants with complete data on all candidate prognostic variables were included. To estimate the risk of selection bias, the baseline characteristics of both included and excluded participants were compared by inspecting all variables’ distributions for significant differences at baseline.

## Results

**Fig. 1:**
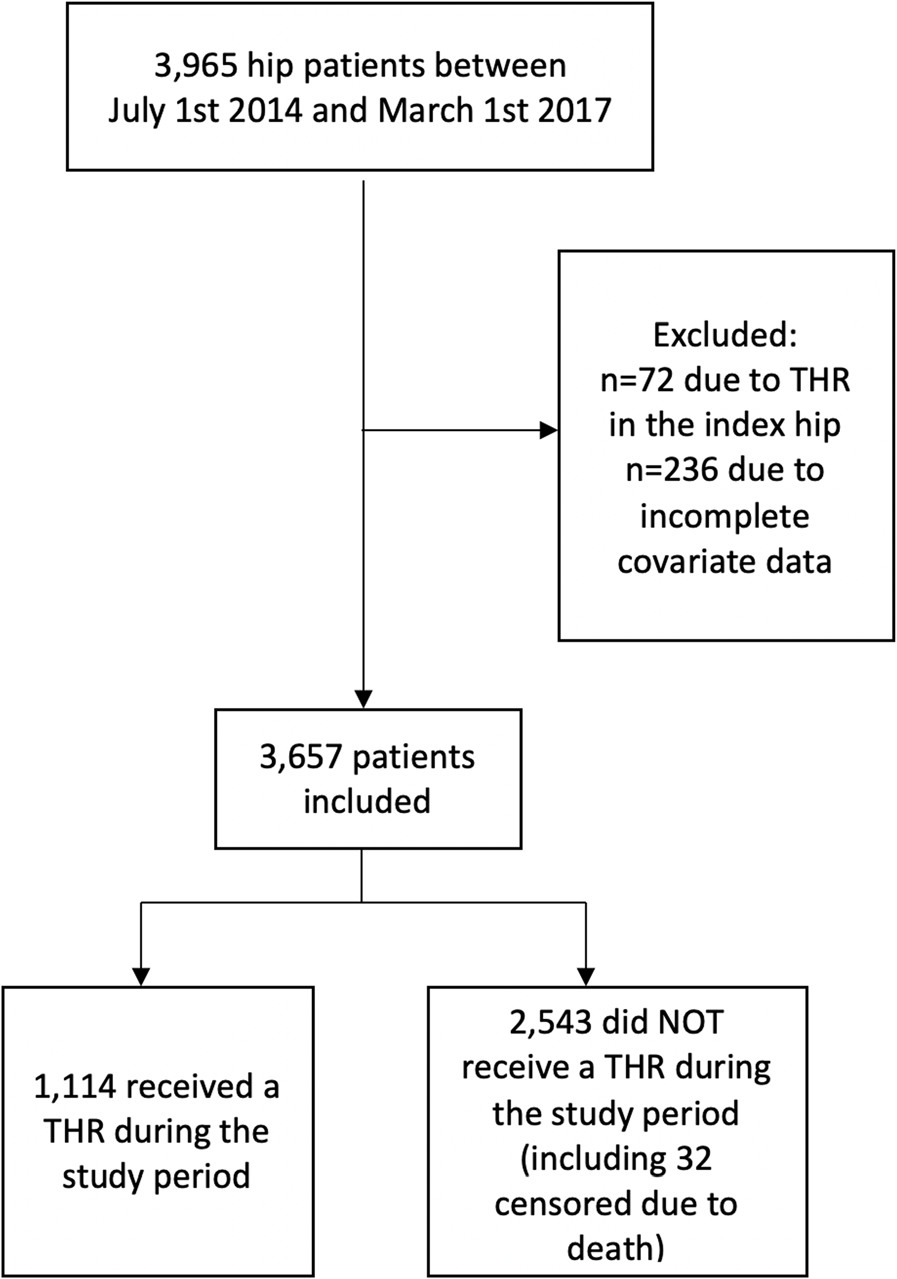
Flow chart of the included participants. THR, total hip replacement

In total, 3965 participants met the inclusion criteria, of whom 308 were excluded due to either previous THR in the index hip (*n* = 72) or missing data in a candidate predictor variable (*n* = 236). No participants emigrated; 32 participants died during the study before any THR (Fig. 1). The excluded participants’ baseline characteristics did not differ from those of the rest of the cohort; additional file 2 shows this in more details.

Baseline characteristics for included participants are listed in Table 1. The mean age was 66.5 years (SD 8.6); 2687 (73%) were women. There were equal numbers having pain in the right and left hip, and the median hip pain on a 0–100 VAS scale was 48 mm (IQR 21).

### Rate of total hip replacement

**Fig. 2:**
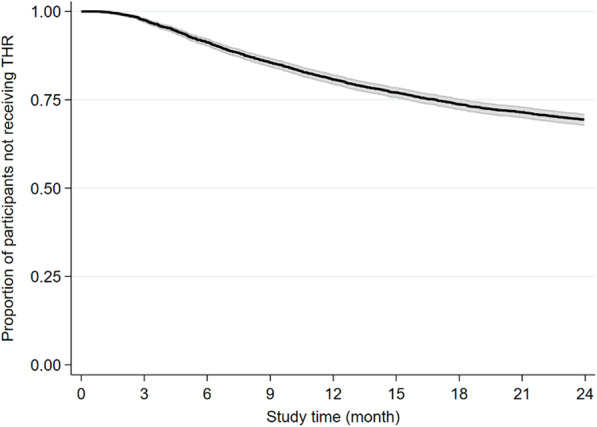
Kaplan-Meier plot with 95% confidence band illustrating the rate of total hip replacement (THR) during the study period

During the 2-year follow-up period, 1114 participants (30%) had a THR (median time to THR 9.6 months, 95% CI 9.0–10.1). Figure 2 illustrates the rate of THR within the study period.

**Fig. 3:**
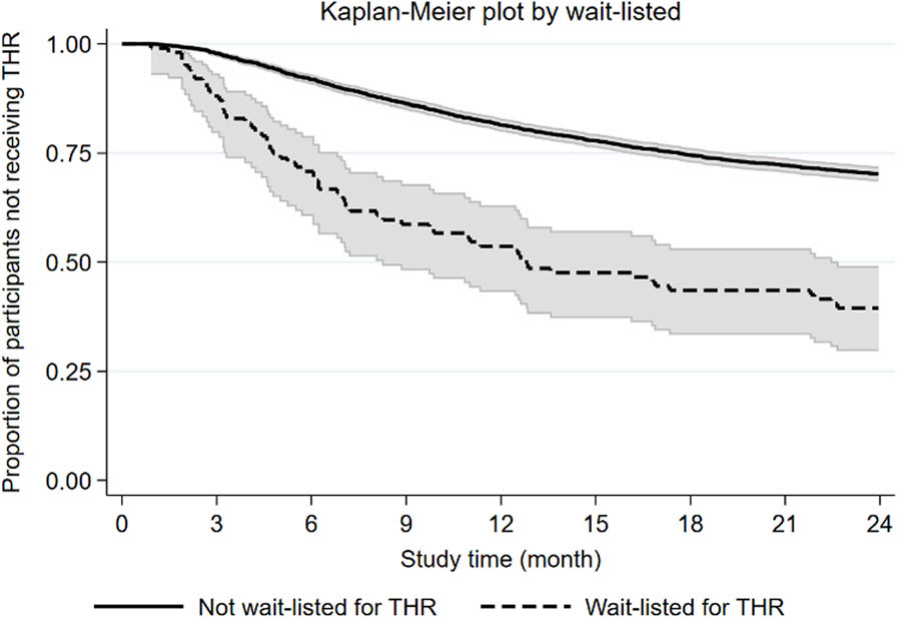
Kaplan-Meier plot with 95% confidence band illustrating the time to total hip replacement (THR) within the study period by THR wait-list status

**Fig. 4:**
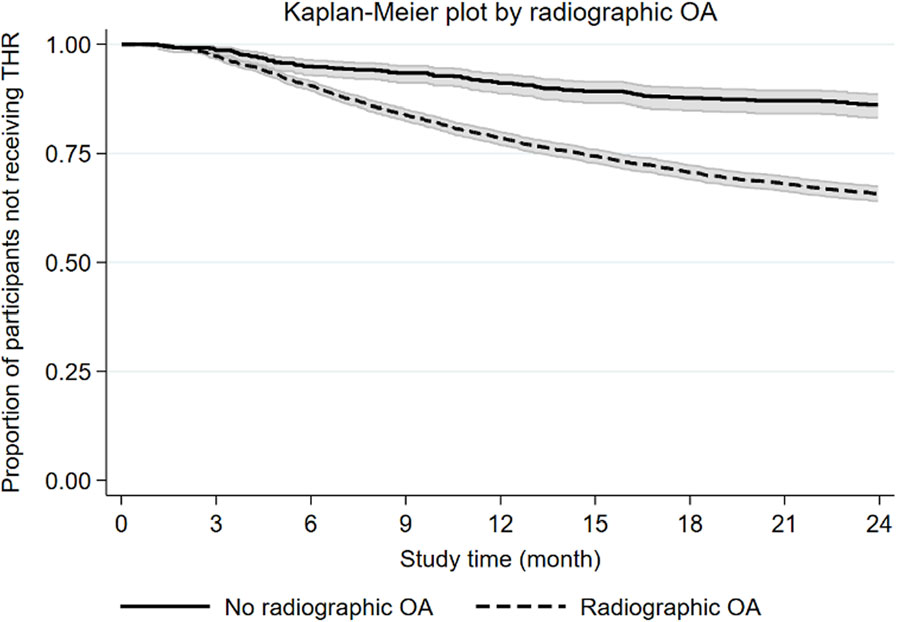
Kaplan-Meier plot with 95% confidence band illustrating the time to total hip replacement (THR) within the study period by presence of self-reported radiographic osteoarthritis (OA)

One hundred participants reported being “wait-listed for THR” at baseline, and of these, 60 (60%) received a THR within 2 years (median time to THR 6.1 months, 95% CI 4.6–7.2). When asked about radiographic hip OA 650 participants reported “no” or “had no x-ray” or “do not know,” and of these, 90 (14%) received a THR (median time to THR 9.7 months, 95% CI 6.7–11.3). Figures 3 and 4 illustrate the rate of THR stratified for “wait-listed for THR” and the presence of “self-reported radiographic OA.”

### Model development

VIFs showed no risk of multicollinearity. The EQ-5D-5L and HOOS QoL had a correlation of 0.7, and EQ-5D-5L was excluded from the model due to collinearity risk. As age was used as the time scale in the model, the proportional hazards assumption was not violated for any of the candidate prognostic factors, and all continuous variables met the assumption of linearity.

The univariate and multivariable relationship between candidate prognostic factors and Time-to-THR are presented as HRs in Table 2. “Fear of joint damage from physical activity,” “living alone,” “30-second stand chair test,” “educational level,” “sick leave,” “Arthritis Self-Efficacy Scale,” and “UCLA score” had a *p* value > .05 and were excluded during the stepwise elimination.

**Table 2:** Univariate and multivariable Cox regression analysis on the relationship between candidate prognostic factors and Time-to-THR

Baseline patient characteristic (candidate prognostic factor)	Unadjusted model		Adjusted model	
	HR (95% CI)	***P*** value	HR (95% CI)	***P*** value
Male (vs. female)	1.24 (1.09-1.41)	< 0.01	1.43 (1.25–1.64)	< 0.01
BMI, kg/m^2^	1.01 (0.99-1.02)	0.26	0.98 (0.97–1.00)	0.01
Smoking (vs. no smoking)	0.83 (0.67-1.03)	0.09	0.70 (0.56–0.87)	< 0.01
Employed (vs. retired)	1.31 (1.07-1.59)	0.01	1.25 (1.02–1.52)	0.03
Use of pain medication the last three months (vs. no pain medication)	1.66 (1.46-1.90)	< 0.01	1.42 (1.23–1.63)	< 0.01
Self-reported radiographic OA (vs. no ROA)	2.81 (2.26-3.48)	< 0.01	2.32 (1.87–2.88)	< 0.01
Comorbidities None One Two Three or more	Reference category 0.96 (0.84–1.10) 0.92 (0.77–1.09) 0.73 (0.57–0.94)	0.59 0.34 0.01	Ref. category 0.90 (0.79–1.04) 0.85 (0.71–1.02) 0.62 (0.48–0.81)	0.15 0.08 < 0.01
Wait-listed for THR (vs. not wait-listed)	2.88 (2.22–3.75)	< 0.01	2.17 (1.66–2.83)	< 0.01
Joint replacement in other hip or knees (vs. no previous THR/TKR)	1.50 (1.26–1.79)	< 0.01	1.44 (1.20–1.72)	< 0.01
Bilateral hip symptoms (vs. unilateral symptoms)	0.73 (0.63–0.84)	< 0.01	0.78 (0.67–0.90)	< 0.01
Number of painful areas during the last 24 h (out of 0–56 possible areas)	0.97 (0.95–0.99)	< 0.01	0.94 (0.92–0.96)	< 0.01
Hip pain (VAS 0–100)	1.01 (1.01–1.2)	< 0.01	1.01 (1.00–1.01)	< 0.01
HOOS QoL score (0–100)	0.97 (0.97–0.98)	< 0.01	0.98 (0.97–0.98)	< 0.01
40 m walk test (m/s)	0.52 (0.43–0.63)	< 0.01	0.64 (0.51–0.80)	< 0.01
30 s chair stand test (number of rises)	0.97 (0.95–0.99)	< 0.01	Excluded^a^	
ASES (10-100)	0.99 (0.99–0.99)	< 0.01	Excluded^a^	
UCLA activity score collapsed to five categories 1–2 3–4 5–6 7–8 9–10	Ref. category 1.32 (0.74–2.36) 1.28 (0.72–2.28) 1.35 (0.76–2.40) 1.16 (0.63–2.14)	0.34 0.41 0.31 0.64	Excluded^a^	
Fear of joint damage from activity (vs. no fear)	1.22 (1.01–1.48)	0.04	Excluded^a^	
Educational level Primary school Secondary school Short-cycle higher Middle-cycle higher Long-cycle higher	Ref. category 1.04 (0.82–1.31) 1.04 (0.85–1.28) 1.05 (0.89–1.25) 1.12 (0.90–1.40)	0.76 0.68 0.56 0.31	Excluded^a^	
Living alone (vs. living with others)	0.95 (0.83–1.09)	0.48	Excluded^a^	
Sick leave more than 1 month (vs. no sick leave or less than a month)	1.25 (0.87–1.81)	0.23	Excluded^a^	

HOOS QoL, Quality of life subscale score from the Hip disability and Osteoarthritis Outcome score (worst to best)

ASES, Arthritis Self-Efficacy Scale (worst to best)

UCLA, University of California, Los Angeles activity score

^a^Excluded = eliminated in the backward stepwise elimination

### Prognostic factors for total hip replacement

Fourteen prognostic factors were statistically significant for receiving THR within the 2 years. Factors associated with a faster rate of THR included being “male” (HR 1.43), being “employed” (HR 1.25), “using pain medication the last three months” (HR 1.42), having “self-reported radiographic hip OA” (HR 2.32), being “wait-listed for THR” (HR 2.17), “previously undergoing joint replacement of the other hip or in the knees” (HR 1.44), and higher “pain intensity” (HR 1.01). Factors associated with a slower rate of THR included faster “walking speed” (HR 0.64), better “hip-related QoL” (HR 0.98), having “three or more comorbidities” (HR 0.62), higher “BMI” (HR 0.98), “bilateral symptoms” (HR 0.78), more “painful areas during the last 24 h” (HR 0.94), and “smoking” (HR 0.70).

As previously described, duration of symptoms was not included as a potential prognostic factor. After fitting the model, we did a sensitivity analysis fitting the model again with duration of symptoms included as a prognostic factor. The univariate hazard ratio for duration of symptoms was 1 (CI 1.00–1.00), and overall, the adjusted model did not change.

### Internal validation and model performance

The bootstrapping procedure revealed low risk of overfitting. The discriminative ability of the model was acceptable with Harrell’s c-index = 0.7 (95% CI 0.6–0.7) [20].

## Discussion

In this cohort study of participants with hip OA enrolled in a supervised education and exercise therapy program, 30% received a THR within 2 years of first enrolling. Interestingly, 40% of those already wait-listed for THR when enrolled had not received a THR after 2 years, suggesting that even those eligible for surgery can change the course towards THR. Of 22 baseline candidate prognostic factors, 14 were statistically significant for receiving THR.

Previous prognostic studies of hip OA from New Zealand, France, and Australia [12, 21, 22] reported prevalence of 2-year THR rates between 37% and 50% compared to the 30% that received a THR within 2 years in our study. Differences in healthcare systems may explain this difference, besides participant selection, and types of non-surgical treatments received. In Denmark, there is easy access to public healthcare services. Moreover, participants in the previous studies had worse baseline hip pain or QoL and longer symptom duration than those in our cohort, which may help explain the higher THR rates. However, although education and exercise therapy are recommended as the first-line treatment [4, 5], median symptom duration of 24 months (IQR 40) in our cohort shows that most participants did not engage in the GLA:D® program until relatively late in their disease course and may have had other types of treatment before enrolling. Van Berkel et al. [23] included first-time presenters with hip complaints in their study of the natural course of early hip osteoarthritis and found that participants were on average 10 years younger and had significantly fewer and milder symptoms compared to the participants in our study suggesting that indeed our participants were not first-time presenters.

As expected, being “wait-listed for THR” at baseline was associated with a faster THR rate within 2 years (HR 2.17). However, it is noticeable that out of the 100 wait-listed, only 60 received a THR within 2 years, given that the waiting time for THR is 1 or 2 months in Denmark. Some who were wait-listed for THR might not have been ready to consent to the surgery right away and chose non-surgical treatment to see whether they could avoid surgery. There is evidence that non-surgical treatment can reduce the need for THR [22] and is feasible in participants eligible for total joint replacement [24]. Future studies should evaluate whether treatment effects or adherence to the program are associated with THR rate during the 2 years follow-up and whether the GLA:D® program or a similar treatment program can delay or maybe prevent THR in participants with hip OA considered eligible for joint replacement.

“Self-reported radiographic OA” was a prognostic factor for receiving THR (HR 2.32). The majority of participants (82%) reported radiographic OA at baseline, indicating that even though hip OA is a clinical diagnosis, most participants who seek care from physiotherapists have had prior radiographs. This is not surprising as routine radiographic evaluation is common despite radiographs providing little value in addition to the clinical assessment in primary care [25, 26]. Our study did not include radiographic severity, but comparable studies [12, 21, 22, 27] have found that more severe radiographic OA is predictive of a higher THR risk. This association probably relates to radiographic, end-stage OA being one of the most commonly applied criteria for recommending THR [28].

Among the physical function and activity measures, “walking speed” was the only prognostic factor included in the model. A faster “walking speed” was a protective factor for THR within 2 years (HR 0.64). Self-reported “Hip pain” (HR 1.01) and “hip-related QoL” (HR 0.98) were also prognostic factors, with more pain and worse hip-related QoL associated with faster rate of surgery within the study period. Previously reports on the prognostic value of pain and physical limitation have been conflicting [6, 29], highlighting the need for further investigation.

The study also found that having “three or more comorbidities,” a high “BMI,” and “smoking” were prognostic factors associated with a reduced rate of THR. These factors are unlikely to be associated with better prognosis in general but have previously been demonstrated to be associated with an increased risk of complications during surgery [28], which could lead surgeons to recommend against THR. The variability in surgeons’ recommendations and practices is an essential contributor to the variability in clinical symptoms among hip participants receiving THR [29]. Thus, especially when THR is interpreted as a proxy for disease progression, it must be kept in mind that multiple factors inevitably influence the decision to perform surgery as well as the prognosis in general.

### Strengths and limitations

Strengths included using surgical codes from complete public registry data as the outcome ensures high validity [9] and the large cohort (*n* = 3657) with a relevant number of cases having the outcome (1114), which made the analysis of a number of prognostic factors possible.

Some limitations are important to mention. Although we included a large number of variables considered to be important for progression to THR, we might have missed important prognostic factors. The decision to perform surgery involves many factors not investigated in this study, i.e., the participants’ willingness to undergo surgery and preference for surgical versus non-surgical treatment, doctors and other health care professionals’ opinions, and organization of the healthcare system. Furthermore, radiographic hip OA was self-reported, which might not be as accurate as an actual radiographic evaluation of hip OA. If radiographs had been available, we would also have been able to determine the importance of the severity of radiographic findings. Finally, the study was conducted in patients seeking primary care physiotherapy, and the results cannot necessarily be generalized to all patients with hip OA.

## Conclusion

In participants with symptomatic hip OA enrolled in a supervised education and exercise therapy program, 30% of the cohort received a THR during the 2-year follow-up period, which was at the lower end of rates reported in previous studies of hip OA. Fourteen baseline prognostic factors for receiving THR were identified, and the results provide knowledge about progression to THR.

Noticeably, 40% of participants wait-listed for THR when entering the program did not receive THR within 2 years.

## Supplementary Information

**Additional file 1.** Overview of registry questions for baseline variables.

**Additional file 2.** Overview of baseline characteristics for included and excluded participants.

## Data Availability

The data that support the findings of this study are available from The Danish National Patient Registry, the Danish Civil Registration System and University of Southern Denmark, but restrictions apply to the availability of these data, which were used under license for the current study, and so are not publicly available. Data are however available from Dr. Roos (eroos@health.sdu.dk) and Dr. Skou (stskou@health.sdu.dk) upon reasonable request and with permission from The Danish National Patient Registry and The Danish Data Protection Agency at University of Southern Denmark.

## Abbreviations

OA: Osteoarthritis
THR: Total hip replacement
GLA:D®: Good Life with osteoArthritis in Denmark
BMI: Body Mass index
UCLA: University of California, Los Angeles Physical Activity Scale
HOOS QoL: Hip disability and Osteoarthritis Outcome Score, the quality-of-life subscale score
ASES: Arthritis Self-Efficacy Scale
EQ-5D-5L: The EuroQoL 5-Dimensions 5-Level questionnaire
NSAID: Non-steroidal anti-inflammatory drugs
VAS: Visual analog scale
IQR: Interquartile range
SD: Standard deviation
VIF: Variance inflation factor
HR: Hazard rate
