# Supplementary material for: Prognostic factors of total hip replacement during a 2-year period in participants enrolled in supervised education and exercise therapy: a prognostic study of 3657 participants with hip osteoarthritis
Source: Arthritis Res Ther. 2021 Sep 7;23:235. doi: 10.1186/s13075-021-02608-6 (PMC8422712; doi:10.1186/s13075-021-02608-6)
Supplement: Supplementary file 1 — Additional file 1. Overview of registry questions for baseline variables. [file 13075_2021_2608_MOESM1_ESM.docx]

Additional file 1. Overview of registry questions for baseline variables

| **Variable** | **Question(s)** | **Answer options** | **Data source** |
| --- | --- | --- | --- |
| 1. Age | Age |  | Calculated from the person-unique personal identification number assigned to all persons residing in Denmark (CPR) and date of first visit |
| 1. BMI | 1. Weight  2. Height |  | Calculated from weight and height  collected by the therapist |
| 1. Sex | Gender | Male  Female | Derived from CPR |
| 1. Hip side | Most affected joint? | Right hip  Left hip | Patient |
| 1. Smoking | Do you currently smoke? | Yes  No | Patient |
| 1. Living situation | Are you living alone or with others? | Living alone  Living with others (partner, family, friends or others) | Patient |
| 1. Sick leave more than one month because of the hip during the last year | Have you been on sick leave because of knee/hip during last year?  and  For how long time have you been on sick leave because of knee/hip during last year? |  | Patient  Calculated from the questions regarding sick leave |
| 1. Educational   level | What is your highest completed education? | 1 Primary school  2 Secondary school  3 Short-term education (under three years after secondary school)  4 Middle-term education (three to four years after secondary school)  5 Long-term education (at least five years after secondary school) | Patient |
| 1. Current employment | What is your current employment? | 1 Employed/student  5 Unemployed  2 On sick leave full time  3 On sick leave part time  7 Early retirement due to low ability to work  6 Self-imposed early retirement  4 Retired | Patient  Coded binary ‘retired’ no matter what kind of retirement or ‘at the labor marked’ regardless of current sick leave or temporary unemployment. |
| 1. Radiographic signs of knee OA | Did x-ray show OA? | Yes  No  No x-ray / do not know | Therapist  Collapsed into radiographic OA present or absent. If “do not know” or “no x-ray performed” was reported it was considered as “no radiographic OA”. |
| 1. Waitlisted for surgery | Is the patient waitlisted for surgery? | Yes  No | Therapist |
| 1. Replaced knee or hip joints | Have you had a joint replacement in hip or knee? | Yes  No | Patient  Except the index joint.  THR in the index joint at baseline is an exclusion criterion |
| 1. Comorbidity | Do you have high blood pressure?  Do you have heart disease?  Do you have a chronic lung disease?  Do you have diabetes?  Do you have ulcers in your stomach or other stomach disease?  Do you have a kidney or a liver disease?  Do you have anemia or other blood disease?  Do you have cancer?  Do you have depression?  Do you have rheumatoid arthritis?  Do you have a neurological disease?  Do you have any other medical disease? | Yes  No | Patient  Collapsed into four categories as none, one, two, three or more. |
| 1. Use of painkillers (paracetamol/ Acetaminophen NSAID/opioids) in the last months | Has the patient used any pain medications including herbal or dietary supplements in the last three months? | We only incluted the following (yes/no):  Paracetamol Acetaminophen/NSAID Morphine  Tramadol  Codeine | Therapist  Calculated from question on the individual medications in three categories:  1. No  2. Yes: Only Paracetamol or Acetaminophen/ NSAID  3. Yes: Morphine, Tramadol, Codeine (everyone in this category did also use analgetics from category 2) |
| 1. Anxious about physical activity | Are you afraid that your joints will be damaged from physical activity and exercise? | Yes  No | Patient |
| 1. Bilateral hip symptoms? | 1. Are your left knee or hips affected? 2. Are your right knee or hips affected? | No  Left knee  Left hip  Right hip  And  No  Right knee  Left hip  Right hip  (more answers are allowed) | Patient  Calculated from questions about symptoms from other joints |
| 1. Number of painful body areas (collected via pain drawing) | 1. On the body chart (front view), please indicate the area(s) of the body where you have felt pain within the last 24 hours 2. On the body chart (rear view), please indicate the area(s) of the body where you have felt pain within the last 24 hours | 1-26 areas (front view) and 1-30 areas (rear view) | Patient  Calculated from pain mannequin front and rear collapsed |
| 1. Baseline pain intensity during the last month (VAS scale 0-100, no pain to worst pain) | I would like you to think about a scale that goes from no pain (0) to worst pain imaginable (100). Using the scale below, please place an ‘X’ through the number that best represents your hip/knee pain during the last month.  (VAS) | 0 No pain  .  .  .  100 Worst pain | Patient |
| 1. Duration of symptoms | How long has the patient had the symptom in most painful joint? | In month | Therapist |
| 1. UCLA - Physical activity score   (from 0-10 worst to best) | What is your current activity level? Consider your activity level during the last 4 weeks. Level 10 is very high and 1 is very low. | 10: Regularly participates in impact sports such as jogging, soccer, handball, badminton, tennis, skiing, heavy labor or backpacking  9: Sometimes participates in impact sports such as jogging, soccer, handball, badminton, tennis, skiing, heavy labor or backpacking  8: Regularly participates in active events, such as bicycling for a long time, golf or hard gymnastics/fitness  7: Regularly participates in active events such as bicycling for a long time, golf or hard gymnastics/fitness  6: Regularly participates in moderate activities such as swimming, bicycling, a long walk or could do unlimited housework or shopping  5: Sometimes participates in moderate activities such as swimming, bicycling, a long walk or could do unlimited housework or shopping  4: Regularly Participates in mild activities such as walking, limited housework and limited shopping  3: Sometimes participates in mild activities, such as walking, limited housework and limited shopping  2: Mostly Inactive or restricted to minimum activities of daily living  1: Wholly Inactive, dependent on others, and cannot leave residence | Patient  For the analysis the original 10 categories were collapsed into 5:  1+2  3+4  5+6  7+8  9+10 |
| 1. HOOS quality of life score   (from 0-100, worst to best) | 1. How often are you aware of your knee problem? 2. Have you modified your lifestyle to avoid potentially damaging activities to your knee? 3. How much are you troubled with lack of confidence in your knee? 4. In general, how much difficulty do you have with your knee? | 1.  Never  Monthly  Weekly  Daily  Constantly  2.  Not at all  Mildly  Moderately  Severely  Totally  3.  Not at all  Mildly  Moderately  Severely  Extremely  4.  Not at all  Mildly  Moderately  Severe  Extreme | Patient  Calculated score from the four questions |
| 1. ASES summary score (from 10 -100, worst to best) | ASES other symptoms score (from 10 -100, worst to best) and  ASES pain score (from 10-100, worst to best) |  | Patient  The mean of the two subscales was calculated |
| 1. EQ-5D-5L score | Mobility  Self-care  Usual activities  Pain / discomfort  Anxiety / depression | Patient has to choose one out of five statements within each of the domains e.g. Mobility:  1 I have no problems in walking about  2 I have slight problems in walking about  3 I have moderate problems in walking about  4 I have severe problems in walking about  5 I am unable to walk about | Patient  Scored using the Danish crosswalk value set (-0.624 to 1; worst to best) |
| 1. Time to complete 40m walking test | 40 meters fast-paced walk test (2 decimal places) | In seconds  (converted to m/sec. | Conducted under the supervision of a physiotherapist. |
| 1. Number of chair stands during 30sec | 30 sec. chair stand test | Number | Conducted under the supervision of a physiotherapist. |

BMI= Body Mass Index, VAS = visual analog scale, UCLA: University of California, Los Angeles Physical Activity Scale (1-10)^1^. Level 10 is very high and 1 is very low, HOOS: Hip disability and Osteoarthritis Outcome Score, the quality-of-life subscale score^2, 3^ (0-100 worst to best), ASES: Arthritis Self-Efficacy Scale^4^. Only subscales for pain and other symptoms were collected and a mean were calculated. Higher scores indicate higher self-efficacy. EQ-5D-5L: The EuroQoL 5-Dimensions 5-Level questionnaire^5^ ^6^ presented as an index value scored using the Danish crosswalk value set.

1. Naal FD, Impellizzeri FM, Leunig M. Which is the best activity rating scale for patients undergoing total joint arthroplasty? Clin Orthop Relat Res 2009; 467: 958-965.

2. Klassbo M, Larsson E, Mannevik E. Hip disability and osteoarthritis outcome score. An extension of the Western Ontario and McMaster Universities Osteoarthritis Index. Scand J Rheumatol 2003; 32: 46-51.

3. Nilsdotter AK, Lohmander LS, Klässbo M, Roos EM. Hip disability and osteoarthritis outcome score (HOOS)--validity and responsiveness in total hip replacement. BMC Musculoskelet Disord 2003; 4: 10.

4. Lorig K, Chastain RL, Ung E, Shoor S, Holman HR. Development and evaluation of a scale to measure perceived self-efficacy in people with arthritis. Arthritis Rheum 1989; 32: 37-44.

5. EuroQol Research Foundation. EQ-5D-5L User Guide 2019. Available from: <https://euroqol.org/publications/user-guides>.

6. Oliveira JS, Hayes A. Clinimetrics: The EuroQol-5 Dimension (EQ-5D). J Physiother 2020; 66: 133.
