# Supplementary material for: Prognostic factors of total hip replacement during a 2-year period in participants enrolled in supervised education and exercise therapy: a prognostic study of 3657 participants with hip osteoarthritis
Source: Arthritis Res Ther. 2021 Sep 7;23:235. doi: 10.1186/s13075-021-02608-6 (PMC8422712; doi:10.1186/s13075-021-02608-6)
Supplement: Supplementary file 2 — Additional file 2. Overview of baseline characteristics for included and excluded participants. [file 13075_2021_2608_MOESM2_ESM.docx]

Additional file 2

Baseline characteristics for included (n=3,657) and excluded participants (n=308).

| Variables | Included | Excluded |
| --- | --- | --- |
| Age, mean (SD) | 66.5 (8.6) | 68.0 (8.9) |
| Sex, female, n (%) | 2,687 (73%) | 230 (75%) |
| BMI, kg/m2, mean (SD) | 26.9 (4.7) | 27.0 (5.1) |
| Hip right side, n (%) | 1,981 (50%) | 180 (58%) |
| Current smoking, n (%) | 353 (9.7%) | 22 (7.1%) |
| Living alone, n (%) | 1,006 (28%) | 90 (29%) |
| Sick leave due to hip problems for more than one month during the past 12 months, n (%) | 93 (2.5%) | 27 (8.8%) |
| Educational level, n (%)  Primary and lower secondary school (9–10 years)  Higher general examination program (12-13 years)  Short-cycle higher education (less than three years more)  Medium-cycle higher education (three to four years more)  Long-cycle higher education (minimum five years more) | 620 (17%)  372 (10%)  665 (18%)  1,550 (42%)  450 (12%) | 62 (20%)  33 (11%)  63 (20%)  109 (35%)  41 (13%) |
| Employment, n (%)  Employed/student  Unemployed  Retired  Self-imposed early retirement  Early retirement due to low ability to work  On sick leave full time or part time | 1,026 (28%)  49 (1.3%)  2,131 (58%)  222 (6.1%)  119 (3.3%)  110 (3.0%) | 70 (23%)  (n<5)  190 (62%)  22 (7.1%)  9 (2.9%)  14 (4.5%) |
| Self-reported radiographic OA, n (%)  Had x-ray with radiographic OA  Had x-ray without radiographic OA  Had no x-ray or do not know | 3,007 (82%)  131 (3.6%)  519 (14%) | 238 (77%)  23 (7.5%)  38 (12%) |
| Wait-listed for THR of the index hip, n (%) | 100 (2.7%) | 11 (3.6%) |
| Joint replacement in the other hip or knees, n (%) | 362 (9.9%) | 40 (13%) |
| Comorbidities, n (%)  None  One  Two  Tree or more | 1,425 (39%)  1,321 (36%)  616 (17%)  295 (8%) | 119 (39%)  106 (35%)  57 (19%)   26 (8%) |
| Pain medication the last three months, n (%)  No use of pain medication  Has used only paracetamol/acetaminophen and/or NSAID  Has used opioids  (everyone using opioids also used paracetamol/acetaminophen and/or NSAID) | 1,308 (36%)  2,033 (56%)  316 (8.6%) | 108 (35%)  166 (54%)  34 (11%) |
| Fear of joint damage from activity, n (%) | 365 (10%) | 26 (8.4%) |
| Bilateral hip symptoms, n (%) | 946 (26%) | 82 (27%) |
| Number of painful areas during the last 24h (0-56 areas marked on a body chart front and rear view), median (IQR) | 3 (3) | 3 (3) |
| Hip pain (average) during the last month (VAS 0-100), median (IQR) | 48 (21) | 48 (35) |
| Duration of symptoms in the index joint, months (n=2770), median (IQR) | 24 (40) | 24 (53) |
| UCLA activity score (1-10), median (IQR) | 6 (3) | 6 (3) |
| HOOS quality of life subscale score, median (IQR) (0-100) | 50 (19) | 44 (19) |
| ASES median (IQR) (10-100) | 68 (26) | 67 (26) |
| EQ-5D-5L score, median (IQR) (-0.624 to 1) | 0.723 (0.110) | 0.694 (0.120) |
| 40 meters walk test (m/sec), median (IQR) | 1.49 (0.43) | 1.41 (0.41) |
| 30 sec. chair stand test (no. of rises), median (IQR) | 12 (5) | 11 (5) |

University of California, Los Angeles Physical Activity Scale (1-10). Level 10 is very high and 1 is very low.

Hip disability and Osteoarthritis Outcome Score, the quality-of-life subscale score (0-100 worst to best).

ASES: Arthritis Self-Efficacy Scale. Only subscales for pain and other symptoms were collected and a mean were calculated. Higher scores indicate higher self-efficacy.

The EuroQoL 5-Dimensions 5-Level questionnaire is a measure of Health-related quality of life and are presented as an index value scored using the Danish crosswalk value set.
